# Valsalva Maneuver Decreases Liver and Spleen Stiffness Measured by Time-Harmonic Ultrasound Elastography

**DOI:** 10.3389/fbioe.2022.886363

**Published:** 2022-05-26

**Authors:** Tom Meyer, Heiko Tzschätzsch, Brunhilde Wellge, Ingolf Sack, Thomas Kröncke, Alma Martl

**Affiliations:** ^1^ Department of Radiology, Charité—Universitätsmedizin Berlin, Berlin, Germany; ^2^ Department of Diagnostic and Interventional Radiology and Neuroradiology, Universitätsklinikum Augsburg, Augsburg, Germany

**Keywords:** ultrasound elastography, time-harmonic elastography, Valsalva maneuver, liver stiffness, spleen stiffness

## Abstract

Ultrasound elastography quantitatively measures tissue stiffness and is widely used in clinical practice to diagnose various diseases including liver fibrosis and portal hypertension. The stiffness of soft organs has been shown to be sensitive to blood flow and pressure-related diseases such as portal hypertension. Because of the intricate coupling between tissue stiffness of abdominal organs and perfusion-related factors such as vascular stiffness or blood volume, simple breathing maneuvers have altered the results of liver elastography, while other organs such as the spleen are understudied. Therefore, we investigated the effect of a standardized Valsalva maneuver on liver stiffness and, for the first time, on spleen stiffness using time-harmonic elastography (THE). THE acquires full-field-of-view stiffness maps based on shear wave speed (SWS), covers deep tissues, and is potentially sensitive to SWS changes induced by altered abdominal pressure in the hepatosplenic system. SWS of the liver and the spleen was measured in 17 healthy volunteers under baseline conditions and during the Valsalva maneuver. With the Valsalva maneuver, SWS in the liver decreased by 2.2% (from a median of 1.36 m/s to 1.32 m/s; *p* = 0.021), while SWS in the spleen decreased by 5.2% (from a median of 1.63 m/s to 1.51 m/s; *p* = 0.00059). Furthermore, we observed that the decrease was more pronounced the higher the baseline SWS values were. In conclusion, the results confirm our hypothesis that the Valsalva maneuver decreases liver and spleen stiffness, showing that THE is sensitive to perfusion pressure-related changes in tissue stiffness. With its extensive organ coverage and high penetration depth, THE may facilitate translation of pressure-sensitive ultrasound elastography into clinical routine.

## Introduction

Ultrasound elastography (USE) can quantitatively characterize tissue stiffness and has a wide range of clinical uses in the diagnosis of various diseases such as liver fibrosis (Srinivasa Babu et al., 2016). In the liver and the spleen, overall tissue stiffness is influenced by fluid properties such as blood volume (Parker, 2015) and pressure (Tzschätzsch et al., 2016; Dittmann et al., 2017). It has already been shown that stiffness of both the liver and the spleen is increased in individuals with portal hypertension (Colecchia et al., 2015; Tseng et al., 2018) and decreases after pressure reduction in the portal circulation following the implantation of a transjugular intrahepatic portosystemic shunt (TIPS) (Guo et al., 2015; Tzschätzsch et al., 2017; Buechter et al., 2018; De Santis et al., 2018). Therefore, assessment of stiffness by elastography is an important tool for the diagnosis and treatment monitoring of portal hypertension. However, the underlying mechanism of how perfusion pressure affects stiffness is not yet well understood.

Similar to portal hypertension, the Valsalva maneuver affects abdominal tissue perfusion pressure. It is defined by the forceful exhalation against a closed airway after a normal or full inspiration (Pstras et al., 2016). Abdominal tissue perfusion pressure is calculated as the difference between mean arterial pressure and intra-abdominal pressure (Dabrowski and Rzecki, 2009). During the Valsalva maneuver, intra-abdominal pressure increases while mean arterial pressure drops slightly below baseline level (Pstras et al., 2016). As a result, abdominal tissue perfusion pressure decreases, which is seen as reduced hepatic venous blood flow in Doppler ultrasound during the Valsalva maneuver (Ipek-Ugay et al., 2017). The effect of the Valsalva maneuver on splenic blood flow has not been investigated so far. We hypothesize that, similar to portal pressure decompression following TIPS implantation, the Valsalva maneuver will lead to softening of both the liver and the spleen. However, previously published studies yielded contradictory results on liver stiffness (Millonig et al., 2009; Horster et al., 2010; Goertz et al., 2012; Ipek-Ugay et al., 2017) and, to the best of our knowledge, the effect of the Valsalva maneuver on spleen stiffness has not been studied before.

The effects of perfusion pressure changes on tissue stiffness have been investigated by both magnetic resonance elastography (Guo et al., 2015; Dittmann et al., 2017) and USE (Tzschätzsch et al., 2016; Tzschätzsch et al., 2017; Buechter et al., 2018; De Santis et al., 2018; Tseng et al., 2018). With its short data acquisition time of 1 s, USE provides a clear advantage over magnetic resonance elastography for assessing the effect of breathing maneuvers on tissue stiffness. Assuming that pressure alterations in highly vascularized organs such as the liver and the spleen affect the whole organ (Parker, 2014; Parker, 2015), we believe that measurement of the stiffness response to pressure should cover the entire organ. However, only a few ultrasound-based elastography techniques can generate stiffness maps of large areas of the liver or the spleen (Ormachea and Parker, 2020). These methods exploit time-harmonic wave stimulation to induce superimposed wave fields from different directions in the abdomen and are therefore referred to as time-harmonic elastography (THE) (Tzschätzsch et al., 2014) or reverberant elastography (Parker et al., 2017). While transient USE or USE based on acoustic radiation force impulse is limited by a small sampling window and restricted penetration depth, THE maps stiffness within the full field of view of up to 13 cm depth (Tzschätzsch et al., 2016). This makes THE particularly useful for examining obese patients with ascites, which is a common finding in diseases with altered abdominal perfusion such as portal hypertension.

The aims of this study are 1) to provide new data that may help overcome the controversy about the effect of the Valsalva maneuver on liver stiffness and 2) to investigate the effects of the Valsalva maneuver on spleen stiffness for the first time.

## Materials and Methods

### Subjects

The study protocol conformed to the guidelines of the Declaration of Helsinki and was approved by the institutional review board (ethics committee) of Charité–Universitätsmedizin Berlin (EA1/276/16). Written informed consent to participation in the experiments and publication of any potentially identifiable data was obtained from all volunteers before inclusion in the study. A group of 17 healthy adult volunteers (5 women) with a median age of 30 years (age range, 23–52 years) were prospectively included in the study in October 2021. Full volunteer characteristics are summarized in Table 1. Exclusion criteria were: frequent alcohol consumption, hepatotoxic medication, diseases affecting the liver and/or the spleen, history of liver and/or spleen surgery or intervention, evidence of disease in B-mode ultrasound, and conditions in which the Valsalva maneuver would have posed a risk such as pregnancy, coronary heart and/or cerebrovascular disease, retinopathy, maculopathy, and sensitivity to sudden changes in blood pressure and/or heart rate.

**TABLE 1:** Characteristics of the volunteers examined in our study.

No.	Age	Sex	Body mass index
	years	m/f	kg/m^2^
1	52	m	25.8
2	30	m	20.8
3	26	m	20.1
4	37	m	20.2
5	33	m	22.0
6	31	m	27.7
7	30	m	23.1
8	38	f	17.5
9	30	m	24.8
10	28	m	23.5
11	28	f	23.8
12	26	f	23.5
13	31	m	33.2
14	29	f	20.7
15	26	m	24.9
16	23	f	22.4
17	38	m	24.7
25th percentile	28		20.8
Median	30		23.5
75th percentile	33		24.8

### Valsalva Maneuver

The most common way to perform the Valsalva maneuver is forceful expiration against a closed airway, which results in an expiratory pressure of approximately 53 mbar (Pstras et al., 2016). In order to standardize the Valsalva maneuver, pressure was measured using a manometer with a mouthpiece tube attached. When the glottis is closed during the expiratory effort, pressures in the mouth and in the lungs may not be the same. However, it may be easier for subjects performing the Valsalva maneuver to keep the glottis closed, as it is more comfortable to keep the predetermined pressure level by elevating the intra-oral pressure alone without at the same time using the expiratory muscles (Looga, 2005). To prevent glottis closure during the Valsalva maneuver, a small hole was punctured into the manometer tube to allow continuous air flow from alveoli during the maneuver.

### Time-Harmonic Elastography

**FIGURE 1:**
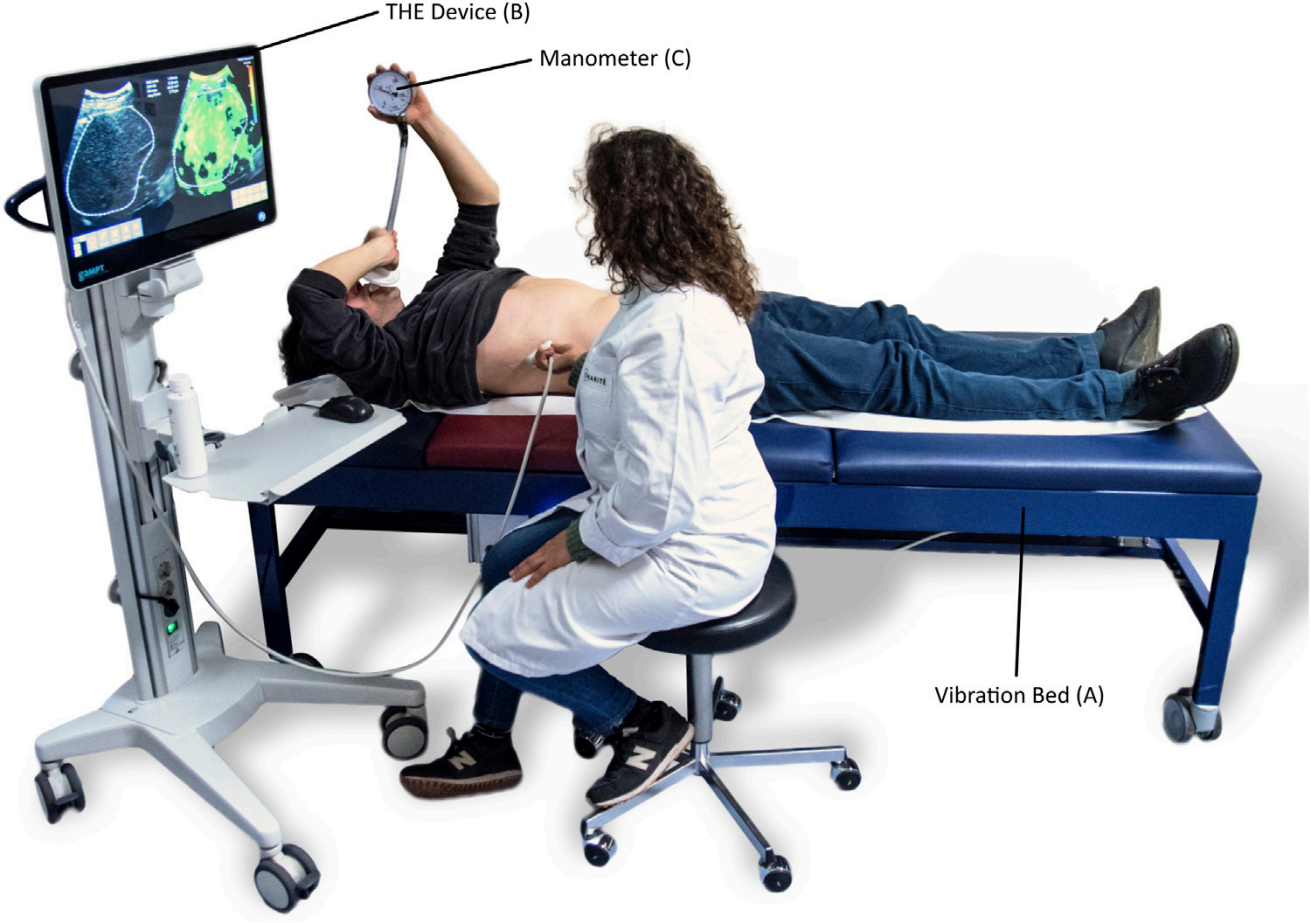
Setup of time harmonic elastography (THE). **(A)** Vibration bed with integrated loudspeaker (underneath the red area of the bed) to induce harmonic vibrations. **(B)** THE device with integrated medical ultrasound system for data acquisition and elastography computer for generation of shear wave speed maps. **(C)** Manometer with mouthpiece to control expiratory pressure during the Valsalva maneuver.

THE was performed with a commercially available elastography system (GAMPT mbH, Merseburg, Germany) consisting of 1) an ultrasound device with a curved-array transducer for anatomic orientation, positioning, and data acquisition, 2) a vibration bed for shear wave generation, and 3) a computer for processing and displaying elastography data. The experimental setup is illustrated in Figure 1. For the examination, the subject was positioned supine on the vibration bed with the organ of interest directly above the vibration generator and either the right arm (for liver measurement) or left arm (for spleen measurement) in maximum extension. The arm maneuver widens the intercostal spaces for better sonographic visualization of the liver and the spleen.

Based on the anatomical B-mode image acquired with a penetration depth of 13 cm, the transducer was positioned to ensure maximum coverage of the organ of interest. The vibration generator induces mechanical multifrequency vibrations in a range of 27–56 Hz. Elastography data were acquired over 1 s. Final shear wave speed (SWS) maps of the liver and the spleen were generated using the *k*-multifrequency dual elastovisco (MDEV) inversion pipeline as described by Tzschätzsch et al. (Tzschätzsch et al., 2016). A region of interest covering the entire visible organ was specified manually based on the anatomical B-mode image, yielding the mean SWS value.

### Imaging Protocol

According to USE guidelines (Dietrich et al., 2017; Săftoiu et al., 2019), all participants were examined in a fasting state, defined as at least 2 h without drinking and eating, by the same experienced operator (AM). The ultrasound transducer was positioned in the right intercostal space for examination of the right liver lobe and in the left intercostal space for imaging the spleen in a longitudinal view. Artifacts and large vessels in the B-mode image were avoided. In each subject, elastograms, first of the liver and then of the spleen, were acquired during breath-hold both at baseline and during the Valsalva maneuver. This procedure was repeated five times. THE measurement was performed at the end of maintaining the Valsalva maneuver for 10–15 s. Each Valsalva maneuver was followed by a short break of 30 s to ensure full recovery of the cardiovascular response (Pstras et al., 2016). Total examination duration per volunteer was approximately 20 min.

### Statistical Analysis

SWS values for baseline and the Valsalva maneuver were calculated as medians over the five repetitions for the liver and the spleen. Statistically significant differences were identified using the Wilcoxon signed rank test. Linear regression analysis was performed to assess the association between baseline SWS and the change in SWS induced by the Valsalva maneuver in the liver and the spleen. All values are displayed as medians with 25 and 75% percentiles. *p* < 0.05 was considered significant. Statistical analysis was performed in R (version 4.1.1; R-Foundation, Vienna, Austria) using R-Studio (version 2021.09.0; RStudio, PBC, Boston, MA).

## Results

**FIGURE 2:**
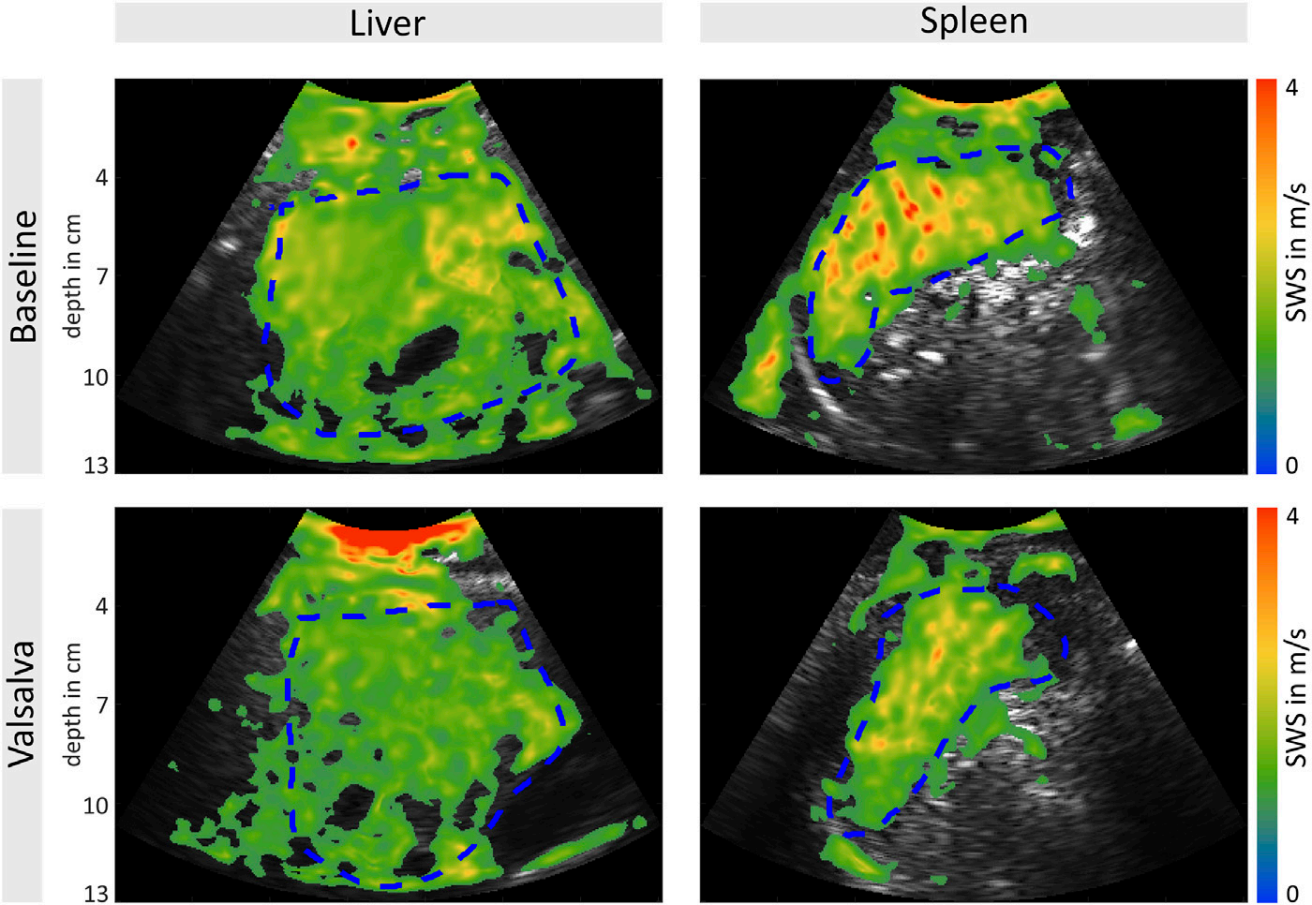
Measurement of liver **(A)** and spleen **(B)** shear wave speed (SWS) under baseline conditions (above) and during the Valsalva maneuver (below). Representative B-mode-images with elastogram overlay showing a decrease in SWS during the Valsalva maneuver. Regions of interest are marked with a dashed blue line.

**FIGURE 3:**
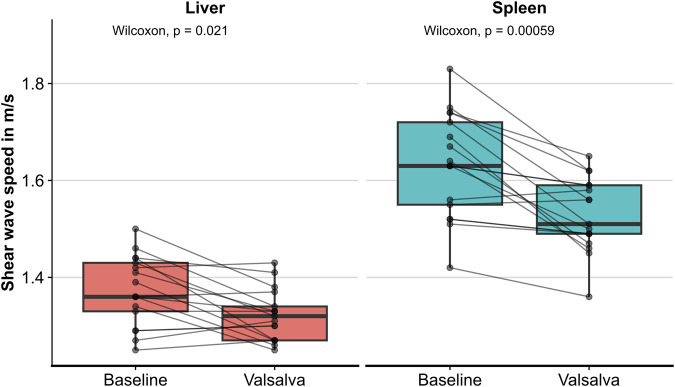
Shear wave speed values of the liver and the spleen under baseline conditions and during the Valsalva maneuver.

SWS values at baseline and during the Valsalva maneuver were obtained from all volunteers for both the liver and the spleen (see representative elastograms in Figure 2). Median baseline SWS was 1.36 m/s for the liver and 1.63 m/s for the spleen (see Table 2). A decrease in liver and spleen SWS during the Valsalva maneuver was observed. The median change in SWS across all volunteers was 0.03 m/s (*p* = 0.021) for the liver and 0.09 m/s (*p* = 0.00059) for the spleen (see Figure 3; Table 2).

**TABLE 2:** Individual and group median values, group 25th and 75th percentiles, and group *p* values of liver and spleen shear wave speed (SWS) at baseline and during the Valsalva maneuver.

No.	Liver			Spleen		
	SWS baseline	SWS Valsalva	ΔSWS	SWS baseline	SWS Valsalva	ΔSWS
	m/s					
1	1.46	1.34	−0.12	1.51	1.49	−0.02
2	1.42	1.43	0.01	1.52	1.49	−0.03
3	1.43	1.32	−0.11	1.75	1.56	−0.19
4	1.29	1.30	0.01	1.83	1.62	−0.21
5	1.25	1.27	0.02	1.63	1.59	−0.04
6	1.39	1.27	−0.12	1.63	1.50	−0.13
7	1.36	1.33	−0.03	1.63	1.59	−0.04
8	1.50	1.38	−0.12	1.64	1.46	−0.18
9	1.36	1.37	0.01	1.72	1.51	−0.21
10	1.41	1.33	−0.08	1.74	1.62	−0.12
11	1.44	1.41	−0.03	1.69	1.45	−0.24
12	1.33	1.33	0.00	1.56	1.58	0.02
13	1.44	1.27	−0.17	1.74	1.65	−0.09
14	1.34	1.25	−0.09	1.55	1.56	0.01
15	1.29	1.30	0.01	1.52	1.49	−0.03
16	1.36	1.26	−0.10	1.67	1.47	−0.20
17	1.27	1.31	0.04	1.42	1.36	−0.06
25th percentile	1.33	1.27	−0.11	1.55	1.49	−0.19
Median	1.36	1.32	−0.03	1.63	1.51	−0.09
75th percentile	1.43	1.34	0.01	1.72	1.59	−0.03
*p* value			0.021			0.00059

For both the liver and the spleen, the magnitude and direction of the observed effect on SWS due to the Valsalva maneuver were not consistent across all 17 subjects. In the liver, 10 volunteers showed a decrease of up to 0.17 m/s, one volunteer showed no change, and six volunteers showed an increase of up to 0.04 m/s. In the spleen, 15 volunteers showed a decrease of up to 0.24 m/s and two volunteers an increase of up to 0.02 m/s (see Figure 3; Table 2).

Four volunteers could not reach and sustain a constant pressure of 53 mbar; however, the change observable during the Valsalva maneuver in these cases was not different from that in the other subjects.

**FIGURE 4:**
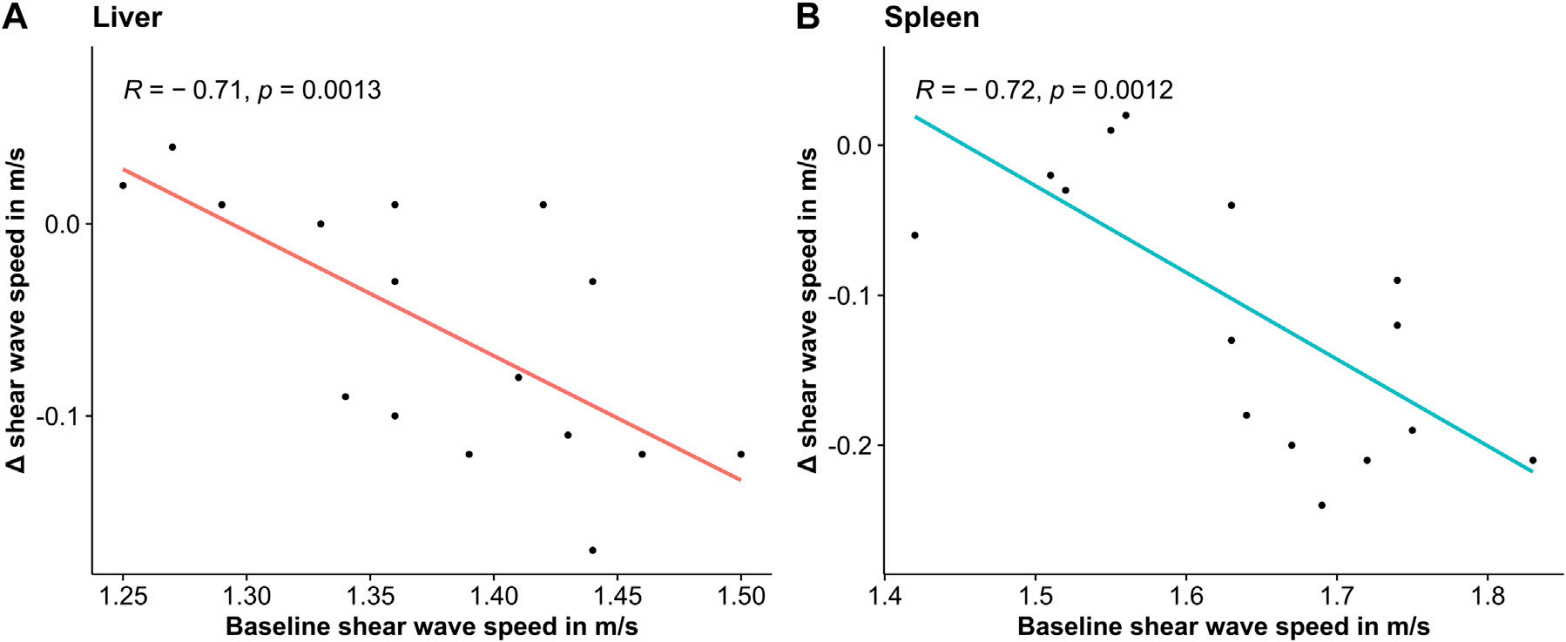
Scatterplot of change in shear wave speed due to the Valsalva maneuver over shear wave speed under baseline conditions together with linear regression fit.

A correlation between SWS under baseline conditions and the change induced by the Valsalva maneuver was observed for the liver (R = −0.71, *p* = 0.0013) and the spleen (R = −0.72, *p* = 0.0012): the decrease in liver and spleen tissue induced by the Valsalva maneuver was more pronounced the higher the baseline SWS was (see Figure 4).

## Discussion

Using a THE device, which allows to acquire full-field-of-view stiffness maps in great depths, we for the first time investigated the effects of the Valsalva maneuver on spleen stiffness in comparison with liver stiffness. In both organs a decrease in stiffness was observed.

In the liver, average softening during the Valsalva maneuver was 2.2%. However, despite standardized execution of the Valsalva maneuver using a manometer, the effect size and direction were not consistent across all subjects. These observations mirror the discrepant results reported in the literature. Compared with our findings, Ipek-Ugay et al. reported a more pronounced decrease in liver stiffness of 13% during the Valsalva maneuver using one-dimensional THE (Ipek-Ugay et al., 2017). Horster *et al.* (Horster et al., 2010) and Goertz et al. (Goertz et al., 2012) observed stiffness changes in both directions, resulting in no significant changes in liver SWS using acoustic radiation force impulse imaging. A 41% increase in liver stiffness was measured by Millonig et al. (Millonig et al., 2009) using transient elastography. Reasons for these discrepancies might be differences in the execution of the Valsalva maneuver, the time delay between start of Valsalva maneuver and measurement and the methods used for stiffness quantification. For example (Ipek-Ugay et al., 2017), used one-dimensional THE, which was repeated 40 times after short (1 s) snapshots within 3 s of Valsalva maneuver. Here, we performed THE at the end of 10–15 s Valsalva maneuver suggesting that the observed effect of softening of liver tissue decreases with time.

In the spleen, we found slightly more pronounced softening (5.2%) than in the liver. Moreover, the group effect in the spleen (decrease in 15 of 17 subjects) was more consistent than in the liver (decrease in 10 of 17 subjects). To the best of our knowledge, there are no other studies that have investigated the effect of the Valsalva maneuver on spleen stiffness. However, TIPS placement also affects perfusion pressure and therefore stiffness in the liver and spleen. Consistent with our results, most studies suggest that spleen stiffness better reflects the change in portal pressure induced by TIPS and may thus be a better measure than liver stiffness to assess the response to TIPS placement (Gao et al., 2012; Guo et al., 2015; Buechter et al., 2018; De Santis et al., 2018; Attia et al., 2019). In healthy volunteers, this might be explained by the fact that the spleen is characterized by a more sponge-like structure with a higher compliance to respond to perfusion pressure changes than the liver (Gao et al., 2012). In patients, spleen stiffness is less severely affected by fibrotic tissue changes in the course of portal hypertension than liver tissue. Since fibrosis reduces mechanical tissue compliance, we expect the spleen to remain more responsive to the Valsalva maneuver with the course of portal hypertension than the liver (Gao et al., 2012).

Our findings support our hypothesis that the Valsalva maneuver lowers tissue stiffness in the liver and the spleen. The link between Valsalva maneuver and tissue stiffness was shown by previous studies which verified that 1) the Valsalva maneuver decreases abdominal perfusion pressure (Pstras et al., 2016) and 2) abdominal perfusion pressure alters abdominal tissue stiffness (Guo et al., 2015; Tzschätzsch et al., 2017; Buechter et al., 2018; De Santis et al., 2018). Possible underlying mechanisms relate to effective-medium stiffness properties, which are altered by vascular softening (Parker, 2014; Parker, 2015) or poroelastic effects such as increased coupling between blood pool and solid tissue (Lilaj et al., 2021). Poroelastic effects in elastography are known to be more relevant in the lower frequency range (McGarry et al., 2015), which might have contributed to the aforementioned discrepancies of results reported in the literature since transient elastography techniques exploit higher frequency bands than THE (Morr et al., 2022). Of note, the baseline stiffness values we measured in our experiment (liver: 1.36 m/s, spleen: 1.63 m/s) are slightly lower than what has been reported in previous studies using THE (Tzschätzsch et. al.: liver: 1.49 m/s, spleen: 2.03 m/s; Heuke et. al.: liver: 1.56 m/s) (Tzschätzsch et al., 2016; Heucke et al., 2019). Reasons might be the small number of subjects in all studies including ours (17–22 years) and the younger age of the subjects we investigated (median age of 30 years versus mean age of 36–39 years).

The absolute effect size of the SWS change due to the Valsalva maneuver correlated with baseline stiffness. Interestingly, we observed similar correlations in both liver and spleen, suggesting that tissue fibrosis is not the reason for these correlations. Instead, one might speculate that different baseline vascular pressures within our cohort influenced tissue stiffness and individual responses to the Valsalva maneuver.

Despite encouraging results, our study has limitations, most notably the small number of healthy subjects and the lack of hepatosplenic Doppler flow parameters for correlation. The current THE setup does not allow simultaneous acquisition of elastography data and Doppler flow parameters. Therefore, sequential acquisition of elastography and Doppler flow data would have required repeated Valsalva maneuvers, which are onerous for subjects and difficult to reproduce. Furthermore, Doppler flow measurement in the spleen during the Valsalva maneuver is technically challenging, depending on individual anatomy and especially with unfavorable sonographic conditions, and standardized measurement would not have been possible in all volunteers.

## Conclusion

In summary, stiffness of both the liver and the spleen measured in healthy volunteers by THE significantly decreased during the Valsalva maneuver. With its high sensitivity to stiffness changes induced by perfusion pressure variation as well as the large coverage of organs in combination with a high penetration depth, THE is of clinical interest for diagnosing and monitoring diseases with altered abdominal perfusion such as portal hypertension.

## Data Availability

The raw data supporting the conclusion of this article will be made available by the authors, without undue reservation.

## Abbreviations

SWS, Shear wave speed; TIPS, Transjugular intrahepatic portosystemic shunt; THE, Time-harmonic elastography; USE, Ultrasound elastography
